# Peptide Controlled Shaping of Biomineralized Tin(II) Oxide into Flower-Like Particles

**DOI:** 10.3390/ma12060904

**Published:** 2019-03-18

**Authors:** Stefan Kilper, Timotheus Jahnke, Katharina Wiegers, Vera Grohe, Zaklina Burghard, Joachim Bill, Dirk Rothenstein

**Affiliations:** Institute for Materials Science, University of Stuttgart, Heisenbergstraße 3, 70569 Stuttgart, Germany; stefan.kilper@gmx.de (S.K.); timotheus.jahnke@imw.uni-stuttgart.de (T.J.); wiegers.katharina@web.de (K.W.); vera.grohe@web.de (V.G.); zaklina.burghard@imw.uni-stuttgart.de (Z.B.); bill@imw.uni-stuttgart.de (J.B.)

**Keywords:** tin(II) oxide, biomineralization, inorganic-binding peptide, phage display

## Abstract

The size and morphology of metal oxide particles have a large impact on the physicochemical properties of these materials, e.g., the aspect ratio of particles affects their catalytic activity. Bioinspired synthesis routes give the opportunity to control precisely the structure and aspect ratio of the metal oxide particles by bioorganic molecules, such as peptides. This study focusses on the identification of tin(II) oxide (tin monoxide, SnO) binding peptides, and their effect on the synthesis of crystalline SnO microstructures. The phage display technique was used to identify the 7-mer peptide SnBP01 (LPPWKLK), which shows a high binding affinity towards crystalline SnO. It was found that the derivatives of the SnBP01 peptide, varying in peptide length and thus in their interaction, significantly affect the aspect ratio and the size dimension of mineralized SnO particles, resulting in flower-like morphology. Furthermore, the important role of the N-terminal leucine residue in the peptide for the strong organic–inorganic interaction was revealed by FTIR investigations. This bioinspired approach shows a facile procedure for the detailed investigation of peptide-to-metal oxide interactions, as well as an easy method for the controlled synthesis of tin(II) oxide particles with different morphologies.

## 1. Introduction

Environmental issues are in the focus of society and politics. To face these questions, materials with tailored morphologies for high functionality in alternative energy storage and generation are needed. Tetragonal tin(II) oxide (tin monoxide, SnO) is, due to its excellent electrical, physicochemical, and optical properties, highly interesting for applications as a p-type semiconductor [1], electrode material for lithium-ion batteries [2], or as a catalyst [3,4] for various organic reactions, e.g., trans-/esterification for the synthesis of bio-diesel. As the shape and size of a material strongly influence its physicochemical properties [5,6], a number of different micro- and nanostructures, such as SnO nanowires [7], nanosheets [8], micro-crosses [9], or micro-flowers [6] have been recently published. Gas-phase and solution-based methods, such as chemical vapor deposition (CVD) [10] or hydrothermal synthesis [11,12] have, been established. In these processes, the reaction is controlled mainly by temperature, pressure, pH value, or concentration of the reactants. 

A high degree of control in the synthesis of inorganic materials is accomplished in the natural process of biomineralization. Because of that, this process is attractive as a model for the targeted development of nanostructures or hierarchically structured assemblies in technical processes. Biomineralization is the formation of inorganic materials by living organisms, giving the opportunity to control the phase and morphology of the hybrid materials by bioorganic molecules, such as proteins [13]. Control over the synthesis is based, *inter alia*, on the specific interaction between the organic and inorganic phase. However, nature’s toolbox on inorganic-binding peptides is limited mainly to materials with restricted technical importance. Therefore, methods such as the phage display (PD) technique are essential for the identification of peptides, specifically those interacting with technically relevant metals, such as semiconductors or insulators like gold (Au) [14], zinc oxide (ZnO) [15], zirconia (ZrO_2_) [16], or titania (TiO_2_) [17]. Even though SnO is a material with a high technical potential, there have been no specifically binding peptides identified up to now. Only peptides interacting with tin(IV) dioxide (SnO_2_) have been reported [18,19]. 

Here, we present the identification of the 7-mer SnO-binding peptide SnBP01 (sequence: LPPWKLK) identified by the phage display technique. The peptides’ affinity to polycrystalline SnO has been determined with a phage-based binding assay. The binding assay shows that genetically modified M13 phages, expressing SnBP01 fused to the N-terminus of the minor coat protein pIII, have a ~60 times higher binding affinity to SnO than the non-modified M13 wild type (wt) phages. On the molecular level, the N-terminal leucine (L) residues of SnBP1 have been determined as one of the interaction sites of the peptide and the SnO. In a hydrothermal SnO synthesis approach, the peptide SnBP01 and variants of it (SnBP01-1: LPPW or SnBP01-2: WKLK) controlled the aspect ratio of the emerging particles. Due to the influence of the peptide and its derivatives on the synthesis, the aspect ratio of the cross-like SnO structures further increase and form flower-like particles. This allows for the synthesis of micrometer-sided SnO particles with a high aspect ratio. 

## 2. Materials and Methods

### 2.1. Phage Display

A 7-mer random peptide library (New England Biolabs Inc., Ipswich, MA, USA) was used in the phage display (PD) technique to identify SnO binding peptides. The random combinatorial library of peptides is fused to the N-terminus of the minor coat protein pIII of the M13 phage. Ten µl of the phage peptide library contains around 100 copies of each 7-mer peptide. The PD technique was conducted following the manufacturer’s manual [20]. Polycrystalline SnO powder (tin(II) oxide 99.9%; 100 Mesh Powder, Alfa Aesar, Haverhill, MA, USA) with an average particle size of ~20 µm was used as a substrate for the PD experiments. The powder was cleaned by suspending 5 g SnO in Milli-Q H_2_O, followed by 10 min centrifugation at 3220 g. Subsequent removal of the supernatant was followed by resuspension in EtOH, and again centrifugation was performed. After three repetitions of the procedure, the powder was dried at 80 °C. For each biopanning procedure, 20 mg of the SnO powder were equilibrated for 10 min in 1 mL Tris-buffered saline + 0.1 % Tween-20 (TBST0.1) in an ultrasonic bath. After centrifugation at 10621 g for 2 min followed by removal of the supernatant, the SnO powder was resuspended in 200 µl TBST0.1. 10 µl of the phage peptide library (~1.2 × 10^11^ phages) were added for 1 h under light agitation at 20 °C. To remove unbound and weakly bound phages, the SnO powder was washed 10 times with 1 mL TBST0.5. For this, first the powder was sedimented by centrifugation at 10621 g for 2 min. Subsequently, the supernatant was removed and the powder resuspended in 1 mL TBST0.5 by gentle vortexing, followed by another centrifugation step. Bound phages were eluted by 1 mL of 0.2 M Glycine-HCl (pH 2.2) supplemented with 1 mg/ml BSA. After 10 min of gentle mixing, the powder is sedimented by centrifugation, and the supernatant is transferred to a new 1.5 mL tube and neutralized by adding 150 µl of 1 M Tris–HCl (pH 9.1). The obtained M13 phages were amplified in *E. coli* ER2738, according to manufacturer’s recommendations. Briefly, 200 µl of an *E. coli* overnight culture are suspended in 20 mL LB media in a 250 mL Erlenmeyer flask, and ~1 mL of the obtained M13 phage eluate was added and incubated for 4.5 h at 37 °C under vigorous mixing. The M13 phage were purified by the addition of PEG/NaCl and subsequent centrifugation steps. To enrich the obtained peptide pool, the complete biopanning procedure was repeated five times by the application of at least ~1.2 × 10^9^ amplified M13 phages. The enrichment was monitored by DNA sequencing of 30 randomly selected M13 phage clones for the fourth and fifth round, respectively. 

### 2.2. Binding Strength Assay

The binding strength of a single peptide sequence was evaluated following a procedure according to Rothenstein et al. [15]. Briefly, a M13 phage clone, expressing only one peptide sequence fused to its minor coat protein (pIII), was amplified, and the purified M13 phages were suspended in 100 µl Tris-buffered saline (TBS). The phage titer was determined in two independent experiment sets. 

A defined amount (2.0 × 10^9^ phages) of an M13 phage clone, expressing a binding peptide or an M13 wt phage, as reference, were incubated for 60 minutes at room temperature with 20 mg SnO particles. Unbound and weakly bound phages were removed from the SnO substrate by washing it 10 times with 1 mL TBST0.5. The bound phages were eluted from the substrate by the addition of 1 mL of 0.2 M glycine–HCl (pH 2.2), supplemented with 1 mg/ml bovine serum albumin (BSA). The titer of the eluted phages was determined according to manufacturer’s manual (New England Biolabs, Inc.). Briefly, constant amounts of *E. coli* bacteria were incubated with dilutions of the phages and plated on LB medium. The plates were incubated overnight at 37 °C. The plaque-forming units (pfu) on each plate were counted. To calculate the phage titer, the number of pfu were multiplied with the dilution factor and the volume of phages. The binding strength, which directly correlates to the phage titer, was determined by three agar plates from two independent experiments, and normalized to a M13 wt phage titer (M13 wt titer = 1). 

### 2.3. Mineralization of Tin(II) Oxide Microstructures

For the synthesis of SnO particles in the presence of the identified binding peptide sequence (peptide01 (SnBP01): LPPWKLK), and its derivatives (peptide01-1 (SnBP01-1): LPPW and peptide01-2 (SnBP01-2): WKLK), stock solutions of 0.2 M SnCl_2_⋅2H_2_O (ACS reagent 98 %, Sigma-Aldrich, Darmstadt, Germany) and 8 mM peptide were prepared in Milli-Q H_2_O. The peptide SnBP01 (7-mer peptide) and the peptide segments SnBP01-1 and SnBP01-2 (both 4-mer peptides) were purchased from a commercial manufacturer (EMC microcollections, Tübingen, Germany). Since in the C-termini of the peptides are blocked when expressed in the phage, the C-termini of the synthesized peptides were inactivated by amidation [21]. The N-terminal amide groups were not blocked. The synthesis of the SnO reference structures was performed according to a modified reaction route inspired by Iqbal et al. [12]. For the reference reaction, 1 mL of mineralization solution was prepared with a final concentration of 30 mM SnCl_2_⋅2H_2_O and 2.6 M NH_3_ (aq) (28 %, VLSI Selectipur, BASF, Ludwigshafen, Germany). For the peptide-mediated synthesis, 30 mM SnCl_2_⋅2H_2_O, 1mM peptide, and 2.6 M NH_3_ (aq) were mixed. The synthesis of SnO particles was performed in 1.5 mL reaction tubes at 60 °C for 24 h. The SnO particles were identified as black particles precipitating at the bottom of the reaction tube. The white-yellow supernatant was removed with a pipette, and the SnO particles were washed in 10 mL Milli-Q H_2_O, and subsequently in 10 mL ethanol. Finally, the particles were dispersed in Milli-Q H_2_O and dried at room temperature. 

### 2.4. Structural Analysis

Structural characterization of the SnO powder samples was performed with scanning electron microscopy (Zeiss Gemini) (Zeiss, Oberkochen, Germany) at 1.5 kV. The SnO powder was placed into a scanning electron microscopy (SEM) sample holder with a conductive carbon adhesive tab. For the phase analysis of the synthesized particles, the powder X-ray diffraction pattern were obtained using a Rigaku SmartLab 3 kW X-ray diffraction system equipped with a copper anode. The samples were measured on an amorphous silicon plate, to avoid any reflexes of the sample holder. Therefore, a small pile of the synthesized powder was put in the center of the silicon plate, which causes an additional texturation of the measured X-ray data because the anisotropic particle shape, and thereof a non-completely random distribution of the particle orientations. 

### 2.5. Fourier-Transform Infrared Spectroscopy

The Fourier-transform infrared spectroscopy (FTIR) spectra of the synthesized SnO particles were recorded using a Bruker Tensor II spectrometer (Bruker, Billerica, MA, USA) equipped with an attenuated total reflection (ATR) sensor (spectral resolution of ± 2 cm^‒1^). The data analysis was performed with OPUS software. The samples for FTIR spectroscopy were only washed with Milli-Q H_2_O, in order to avoid denaturation of the surface attached peptides. 

## 3. Results and Discussion

### 3.1. Phage Display

For the identification of SnO-binding peptides, a random 7-mer peptide library, displaying ~10^9^ different amino acid sequences, was used. The selection process by phage display was comprised of four to five biopanning rounds, each with four subsequent steps: (1) binding of the phages to the substrate, (2) washing to remove weakly bound phage clones, (3) elution of the bound phages by pH shift of the solution, and (4) amplification of the binding phage clones in *Escherichia coli* (*E. coli*) bacteria (Figure S1) [20]. For an inorganic target, commercially available SnO powder with an average particle size of ~20 µm was used (Figure S2). In total, 50 clones were isolated from the random peptide library: 27 clones after the fourth and 23 clones after the fifth biopanning round. From the 50 phage clones, 13 different peptide sequences were identified. The most enriched peptide was peptide SnBP01, with the amino acid sequence LPPWKLK. Moreover, 12 additional SnO-interacting peptides were selected from the peptide library, however in much less abundance (Table 1). 

In order to specify the interaction sites in the peptide, i.e. to identify the interacting amino acid residues, the frequency of single amino acids in the isolated peptides were compared to the amino acid frequency in the native peptide library (Table 2). The enrichment or depletion of an amino acid is a first indication of whether these amino acids might play a role in the organic–inorganic interaction. For this, the multiplication factor was calculated, which is the quotient of the frequency of an amino acid in the confined peptide pool and the initial frequency of the amino acid in the native peptide library [15]. Amino acids with a multiplication factor of more than 1.25 are defined as being enriched—between 0.75 and 1.25 the amino acids occur in similar frequencies, and below 0.75 the amino acids are depleted. The most enriched amino acid was the basic histidine (H), which occurs 3.11 times more often than in the original library. The basic amino acids arginine (R) and lysine (K) were also enriched by a factor of 1.54 and 1.53, respectively. Beside the positively charged amino acids, the non-polar amino acid leucine (L) had a multiplication factor of 1.60. Contrasting the enrichment of positively charged amino acids, the negatively charged amino acids aspartic acid (D) and glutamic acid (E) were depleted. 

The isoelectric point of SnO is between pH 5.2 and pH 6.6 [22]; hence the SnO particles are negatively charged at pH 7.5, where the phage display was performed. Therefore, the enrichment of the positively charged amino acids, in addition to the depletion of negatively amino acids in the selected peptides, indicates that electrostatic interactions are one of the major driving forces for the peptide–SnO binding. 

### 3.2. Binding Assay

**Figure 1:**
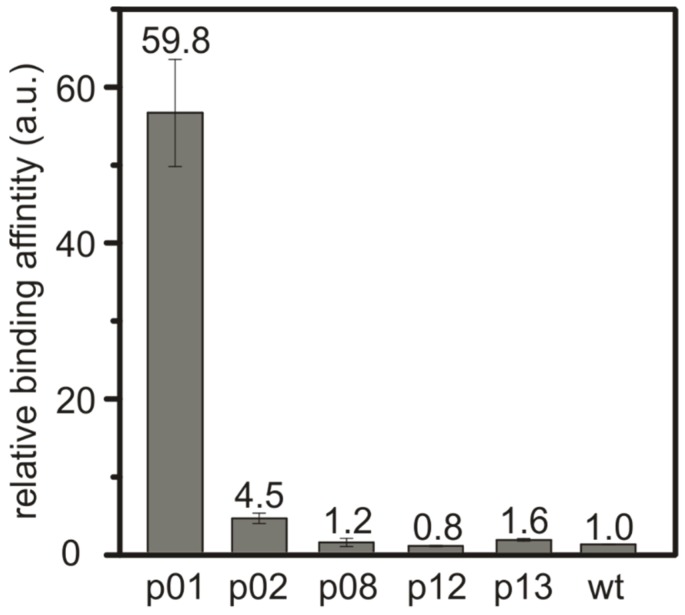
Relative binding strength of the M13 phage clones towards crystalline SnO powder with an average particle size of ~20 µm. The phage clones presenting SnBP01 show a much higher binding affinity towards the SnO surface compared to the other selected peptides or unspecific binding M13 wild-type (wt) phages.

The binding behavior of the identified peptide sequences was investigated for the three most abundant peptides: SnBP01, being LPPWKLK; peptide02 (SnBP02), being WSLSELH; and peptide08 (SnBP08), which is LHRHANL. In addition, peptide12 (SnBP12)—VGKTHAD—and peptide13 (SnBP13)—FPLHELR—which were selected due to a possible binding motif consisting of a charged–uncharged–charged amino acid sequence [23], were investigated. In order to determine the binding strength, M13 phage clones expressing only one of the selected peptides were bound to equal amounts of the SnO powder at pH 7.5 (Figure S2). In previous investigations, we showed that the affinity of inorganic-binding peptides is reflected very well by this phage-based assay [15]. The SnO particles were washed by vigorously mixing in water, to remove the weakly bound phages. This washing step was repeated 10 times, then the phages were eluted and the phage titer was determined. The titer provided a direct measure of the interaction, since it correlates to the relative binding strength of the eluted peptide. The phage titer of the M13 wild-type (wt) phage was used as a reference for the normalization of the binding values [15] (Figure 1). The relative binding affinity of the M13 phage clone expressing the peptide SnBP01 was found to be ~60 times higher than for the M13 wt phage. This reveals a direct correlation of the peptide frequency in the phage display and the binding strength. All other peptides in the binding assay did not show a drastic increase in the binding affinity. 

### 3.3. Mineralization of tin(II) oxide microstructures

**Figure 2:**
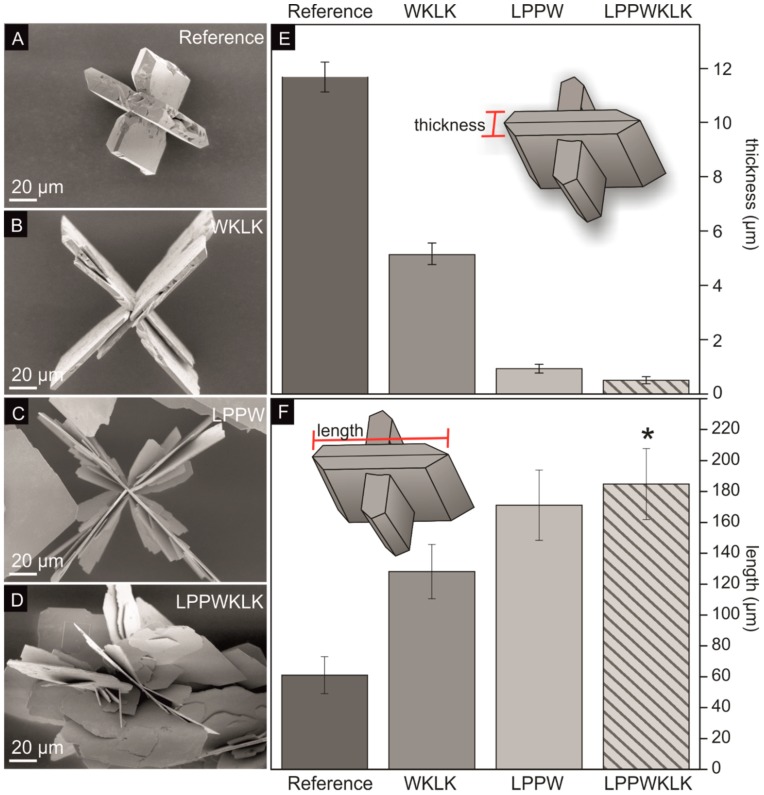
Results of the SnO microstructure synthesis in the presence of the peptide SnBP01 and the peptide segments p-01-1 and SnBP01-2. Scanning electron microscopy (SEM) images of the different SnO morphologies synthesized with (**A**) no peptide, (**B**) SnBP01-2, (**C**) SnBP01-1, and (**D**) SnBP01. The corresponding platelet thickness of the structures is shown in (**E**), and the platelet length in (**F**). * Due to the irregular structure of the flower-like SnO particles, the longest continuous platelet of each particle was measured.

The influence of the binding peptide SnBP01 on the mineralization of tin(II) oxide microstructures was investigated. As a result, the SnO synthesis procedure of Iqbal et al. [12] was modified for moderate synthesis temperatures (60 °C), to prevent denaturation of the peptides. First, SnCl_2_⋅2H_2_O was dissolved in ddH_2_O, and NH_3_ (aq) was added, causing the precipitation of tin hydroxide [24,25]. During this process, the pH of the solution increased to 10.8. After 24 h incubation at 60 °C, black particles became visible. These particles exhibited cross-like SnO structures with a platelet aspect ratio of ~5.2 (Figure 2A, Figure S3, and Table 3). The cross-like SnO particles were formed by twinned platelets with the crystallographic surface planes {001} and {110}, where the larger surface is usually assigned to the {001} planes, which have the lowest surface energy [12]. The platelet aspect ratio is defined as the quotient of the thickness (Figure 2E) and the length (Figure 2F) of one individual platelet. Furthermore, to investigate the influence of the SnO-binding peptide on the observed SnO morphology, the seven-amino-acid-long SnBP01 peptide (LPPWKLK), as well as the N-terminal SnBP01-1 (LPPW), and C-terminal part SnBP01-2 (WKLK) (Figure S4), were applied in mineralization reactions. The peptides were purchased from a commercial manufacturer (EMC microcollections, Tübingen, Germany). In the phage display approach, the peptide library is fused with the peptides’ C-termini to the minor coat protein p3. Therefore, the C-terminal carboxy group of the peptide cannot contribute to the binding. Only the N-terminal amide group is freely accessible for interactions. Therefore, the C-terminus of the synthesized peptides was blocked by amidation [21], and the N-terminus was not blocked. It was found that the morphology of the synthesized SnO particles was strongly influenced by the addition of the peptides. The experiments were performed twice, and the measurement of the particle dimensions (i.e. thickness and length) are based on a minimum of six measurement values. In particular, the addition of peptide segment SnBP01-2 (WKLK) significantly increased the aspect ratio of the cross-like SnO platelets to 25.0 (Figure 2B,E,F; Figure S5; and Table 3) compared to the 5.2 ratio of the reference sample without a peptide. The aspect ratio was further increased to 183.5 by the peptide segment SnBP01-1 (Figure 2C,E,F; Figure S6; and Table 3). The major changes in particle morphology showed the full peptide SnBP01 (LPPWKLK) causing flower-like structures with a platelet aspect ratio of 364.5 (Figure 2D–F; Figure S7; and Table 3). It is noteworthy that the pH of the mineralization solution was preserved during the synthesis. Moreover, the peptides (each at 1 mM) did not have a significant influence on the pH value of the reaction; thus, a pH-induced effect of the morphology changes can be excluded. 

**Figure 3:**
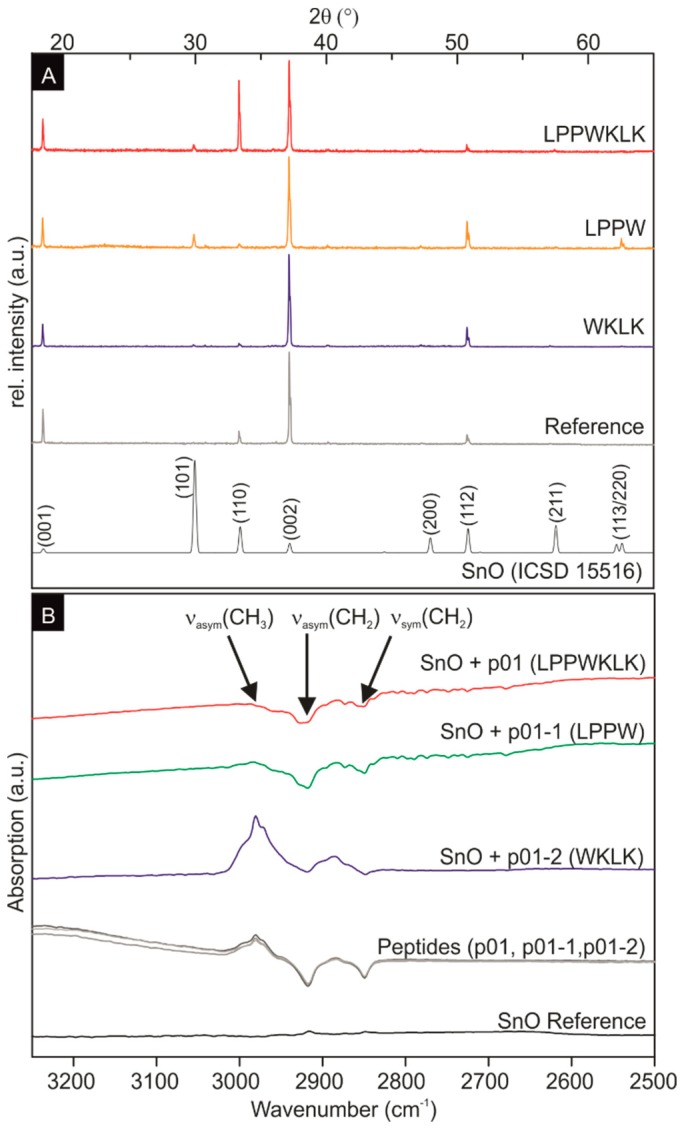
(**A**) SnO powder X-ray diffraction data. The powder X-ray data of crystalline SnO (ICSD 15516) [26] served as a reference. The strong texturation of the measured X-ray spectra is caused by the platelet shape and sample preparation for the measurement. (**B**) FTIR spectra of the SnO reference without peptides (black), the three peptides (SnBP01, SnBP01-1, SnBP01-2) in solution (grey curves), the SnO structures with SnBP01-2 (WKLK) (blue), the SnO structures with SnBP01-1 (LPPW) (green), and the SnO structures with SnBP01 (LPPWKLK) (red).

The powder X-ray measurements of the SnO, mineralized in the presence and absence of peptide additives, showed tetragonal SnO for all samples (Figure 3A). The strong *c*-axis texturation is most probably caused by the platelet shape, preventing a random particle orientation of the powder sample. The large influence of the peptides on the platelet thickness indicate that there is a preferential attachment of the peptides on the {001} planes, which leads to a strong reduction of the growth rate in the [001] direction. 

Accordingly, it is concluded that the addition of peptides enhanced the anisotropic growth of SnO particles. This effect might be attributed either to the concentration of the additives or the strength of the organic–inorganic interaction. Since the molar concentration of all three peptides—SnBP01, SnBP01-1, and SnBP01-2—was constant (1 mM), the influence on the growth can be excluded. With respect to the interaction strength between the peptides and the SnO particles, the peptide SnBP01 and its subunit SnBP01-1 lead to an enhanced anisotropic growth compared to the subunit SnBP01-2. It is noteworthy that the peptide segment SnBP01-2 showed a minor effect on the SnO mineralization, although it contains two lysine (K) residues for potential electrostatic interactions with the SnO surface. However, the high pH of the mineralization solution (pH > 11) might lead to uncharged lysine residues, since the pK_a_ value of lysine is 10.8 [27]. Therefore, electrostatic interactions between lysine and SnO may not be favored. The conclusion is that the various morphologies induced by the three peptides are most likely a result of the interaction strength between peptide and inorganic phase. For non-specific interacting additives, like polyvinyl pyrrolidone (PVP) and gelatin, SnO morphologies were only manipulated by the different additive concentrations [25,28]. 

For the investigation of the binding on the molecular level of the peptide to SnO, Fourier transform infrared (FTIR) spectroscopy studies were performed. Infrared spectroscopy allows the direct and label-free determination of binding events between peptides and inorganic substrates [29,30]. Due to the presence of ammonia in the synthesis, the signals of nitrogen-containing functional groups were observed in all IR spectra, from 4000 cm^‒1^ to 500 cm^‒1^ in all samples (including the SnO reference sample without peptide) (data not shown). Therefore, the FTIR measurements were focused on the spectral range between 3250 cm^‒1^ to 2500 cm^‒1^ (Figure 3) to monitor signals of the CH_2_ and CH_3_ groups of the peptides [29]. Moreover, the synthesized SnO did not possess IR activity in this spectral range (Figure 3B black curve). In solution, the three peptides only (SnBP01, SnBP01-1, and SnBP01-2) display the symmetric (ν_sym_) and asymmetric (ν_asym_) stretching of CH_2_ at 2850 cm^‒1^ and 2917 cm^‒1^, as well as the ν_asym_ of CH_3_-groups at 2980 cm^‒1^, respectively (Figure 3B, grey curves). These signals were also detected in the spectra of SnO particles, which were synthesized in the presence of the peptides. Most interesting, in case of SnBP01 and SnBP01-1, the relative intensity of ν_asym_ for CH_3_ is strongly reduced, while the relative intensities of ν_sym_ and ν_asym_ for CH_2_ were not affected. This can be understood from the fact that of all the investigated amino acids, only leucine (L) has a CH_3_ group. The signal reduction can be attributed to an interaction [31] of a functional group with an SnO particle. Therefore, the binding can be assigned to only the N-terminal leucine with the SnO surface. The signal intensity of the leucine residue of the peptide SnBP01-2, which is internally located, did not change in binding. However, not only the position of leucine at the N-terminus accounted for the binding. Obviously, the neighboring amino acids also influence the interaction, since peptide 08 (LHRHANL), which had an N-terminal leucine, did not show enhanced binding. One can speculate that in SnBP01 and SnBP01-1, the leucine (L) is followed by two proline (P) residues (Figure S7). Proline has a unique influence on the conformation of a peptide, restricting the free rotation of preceding amino acid residues [32], and might therefore advantageously expose leucine. It can be deduced that the main interaction of the peptides SnBP01 and SnBP01-1 with the SnO surface is mediated by the N-terminal leucine residue, which might be accessible due to the conformational restrictions caused by the neighboring proline residues. 

## 4. Conclusions

The bioinspired synthesis route is appealing, due to the generation of bioorganic templates with specific structural and chemical interfaces for the controlled deposition of inorganic components. In particular, inorganic-binding peptides play an important role in the control of the crystal formation. Here, the tin(II) oxide (SnO) binding peptide SnBP01 with the amino acid sequence LPPWKLK was identified by phage display, and applied to morphology control during material synthesis. The peptide SnBP01, genetically engineered on M13 phages, conveyed an ~60 times higher binding strength towards SnO compared to unmodified M13 wt phages. The specific interaction allows for the modification of the SnO morphology in the used synthesis approach. The peptide SnBP01 increased the aspect ratio of the mineralized SnO platelets, and changed the morphology from cross-like to flower-like structures. The interaction of the organic and inorganic phase was ascribed to the N-terminal leucine residue of the SnBP01 peptide, as well as a shortened derivative of this peptide. Moreover, by the comparison with other selected peptides, it was observed that the neighboring proline residues of the leucine enhance the binding capability, probably due to their influence on the peptide conformation. This study shows that inorganic-binding peptides control the aspect ratio and morphology of mineralized inorganic particles, mainly by their specific interactions and not their concentration. This bioinspired approach also has a broad impact with regard to controlling and shaping the morphology and structure of other technologically relevant oxides, for applications where a high surface area is required. 

## Figures and Tables

**Table 1:** Peptides binding to SnO after the fourth and fifth biopanning round.

Peptide	Sequence	Frequency Fourth Round	Frequency Fifth Round	Isoelectric Point (pI) ^a^	Charge, pH 7.5 (a.u.) ^b^
01	LPPWKLK	11/27	17/23	10.00	1.8
02	WSLSELH	4/27	2/23	5.5	-1.1
03	IGASVHR	1/27	0/23	10.00	0.9
04	AHHLKVS	1/27	0/23	9.06	0.9
05	NHPLYNR	1/27	0/23	9.06	0.8
06	ALEHTSR	1/27	0/23	7.19	-0.1
07	HPAIRPP	1/27	0/23	10.00	0.9
08	LHRHANL	2/27	1/23	10.00	0.9
09	SSNQFHQ	1/27	1/23	7.19	-0.1
10	KVPGHQQ	1/27	0/23	9.06	0.8
11	TLAPRTA	1/27	0/23	10.00	0.8
12	VGKTHAD	0/27	1/23	7.19	-0.2
13	FPLHELR	0/27	1/23	7.19	-0.1

* Note: ^a^ the isoelectric point (pI) and ^b^ the charge (a.u.) at pH 7.5 of the unfolded peptide were calculated with Protein Calculator v3.4 (http://protcalc.sourceforge.net).

**Table 2:** Amino acid frequencies in SnO-binding peptides, compared to the native peptide library.

Amino Acid	Multiplication Factor *	Type	Frequency
H	3.11	basic	enriched
L	1.60	nonpolar	
R	1.54	basic	
K	1.53	basic	
A	1.20	nonpolar	Not affected
Q	1.20	polar	
W	1.15	aromatic	
P	1.11	nonpolar	
N	1.01	polar	
F	0.93	aromatic	
S	0.91	polar	
I	0.82	nonpolar	
E	0.74	acidic	depleted
V	0.74	nonpolar	
G	0.72	nonpolar	
T	0.38	polar	
Y	0.20	aromatic	
D	0.19	acidic	
C	0	polar	
M	0	polar	

* Compared to the native peptide library (New England Biolabs), different frequencies of occurring amino acids in the identified peptide sequences are found. The multiplication factor gives the ratio between occurrence and expected amino acid numbers with respect to the native library. Peptides with a multiplication factor of more than 1.25 are defined to be enriched, between 0.75 and 1.25 the amino acids occur in similar frequencies, and below 0.75 the amino acids are depleted [15].

**Table 3:** Thickness, length, and aspect ratio of the synthesized SnO structures.

Sample	Thickness (µm) *	Length (µm) *	Aspect Ratio (Length/Thickness)
Reference	11.67 ± 0.55	61.10 ± 12.00	5.2
WKLK	5.13 ± 0.36	128.10 ±17.53	25.0
LPPW	0.93 ± 0.16	171.16 ± 22.80	183.5
LPPWKLK	0.51 ± 0.13	184.83 ± 22.95 **	364.6

* The measurement of the thickness and length of SnO platelets are based on minimum of six measurement values. ** The length of these particles is based on the longest continuous platelet of a particle.

## Supplementary Materials

The following are available online at https://www.mdpi.com/1996-1944/12/6/904/s1, Figure S1: Schematic description of the phage display technique. Figure S2: Structural characterization of the commercial SnO powder. Figure S3: Overview SEM images of the reference particles synthesized without peptides. Figure S4: Schematic description of the construction of three solid-state synthesized peptides, based on the sequence of SnBP01 for the mineralization study of tin(II) oxide microstructures. Figure S5: Overview SEM images of the SnO particles synthesized with of 1 nM peptide SnBP01-2 (WKLK). Figure S6: Overview SEM images of the SnO particles synthesized with of 1 nM peptide SnBP01-1 (LPPW). Figure S7: Overview SEM images of the SnO particles synthesized with of 1 nM peptide SnBP01 (LPPWKLK).
