# Supplementary material for: Peptide Controlled Shaping of Biomineralized Tin(II) Oxide into Flower-Like Particles
Source: Materials (Basel). 2019 Mar 18;12(6):904. doi: 10.3390/ma12060904 (PMC6470704; doi:10.3390/ma12060904)
Supplement: Supplementary file 1 [file materials-12-00904-s001.pdf]

materials

Supplmentary Information

Peptide controlled shaping of biomineralized tin(II) oxide into flower-like particles

Stefan Kilper [1], Timotheus Jahnke [1], Katharina Wiegers [1], Vera Grohe [1], Zaklina Burghard [1], Joachim Bill [1] and Dirk Rothenstein [1,*]

[1] Institute for Materials Science, University of Stuttgart, Heisenbergstraße 3, 70569 Stuttgart, Germany;
* Correspondence: dirk.rothenstein@imw.uni-stuttgart.de; Tel.: +49 711 685 61938

Received: date; Accepted: date; Published: date

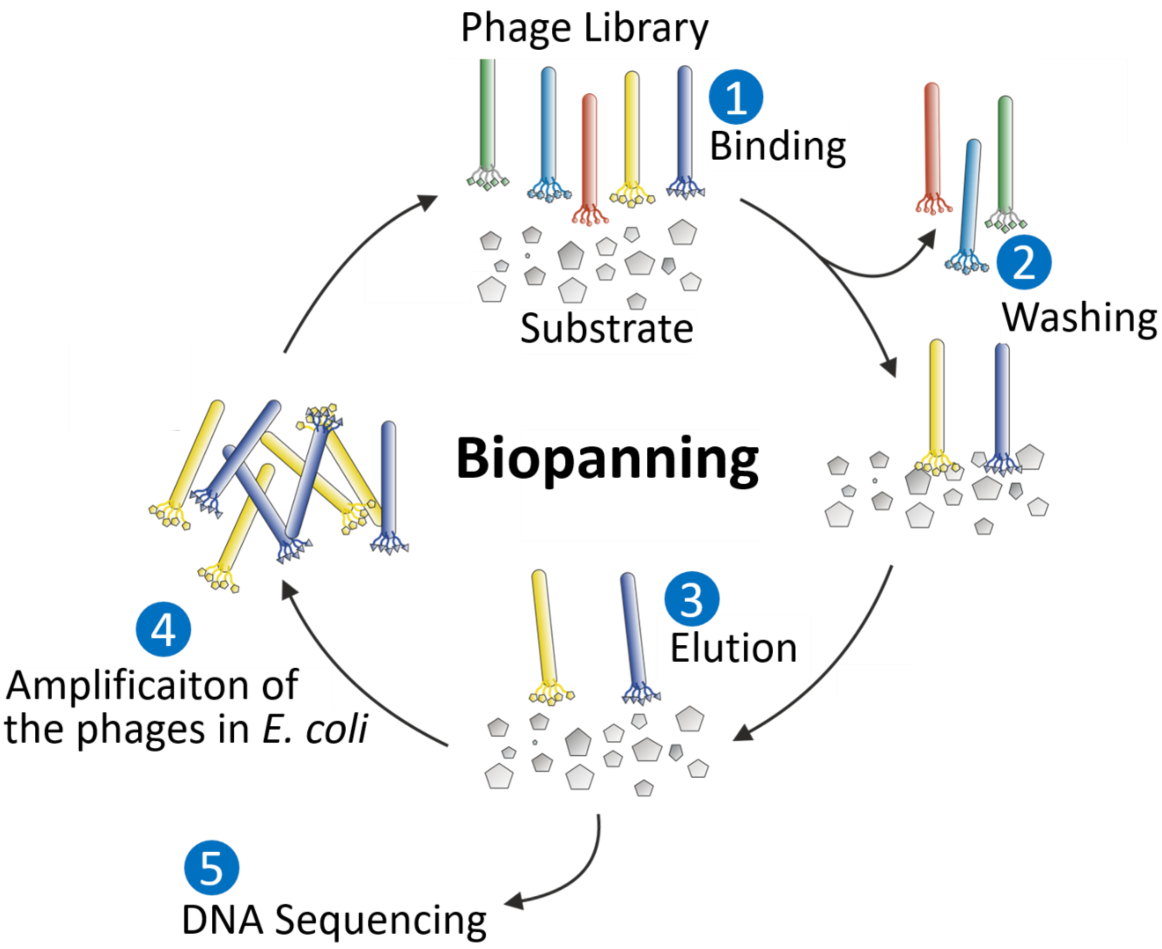

Figure S1. Principle of the phage display strategy for the isolation of SnO-binding peptides. Genetically engineered M13 phages express a peptide library as fusion protein with the pIII minor coat protein (Phage library). The phage-expressed peptide library is incubated with the inorganic target substrate (1). Non-binding phages are eliminated from the peptide pool by washing with appropriate buffers systems (2). Strongly bound phages are eluted by a pH shift (3). To increase the binding specificity of the peptides the eluted phages are amplified in *E. coli* (4) and the steps 1 to 3 are repeated, this procedure is called biopanning. The binding peptides are identified by DNA sequencing of the corresponding DNA section.

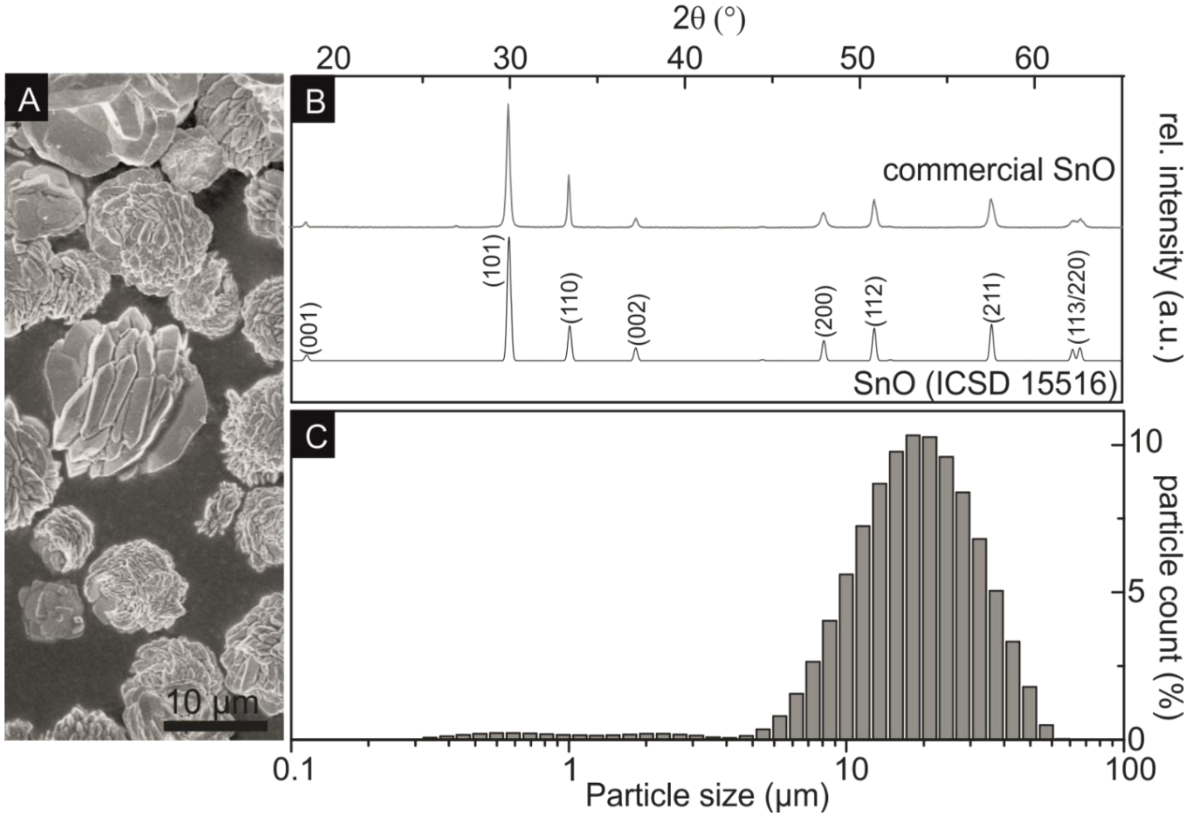

Figure S2. Structural characterization of the commercial SnO powder. A) SEM image of the +commercial SnO particles used for PD and Binding Assay. (B) Powder x-ray diffractogram of the commercial SnO powder. The powder x-ray data of the crystalline SnO (ICSD 15516)[26] used as reference. C) Particle size distribution. The particle size distribution was measured using a Mastersizer 2000 APA5005 (Malvern Instruments GmbH, Germany).

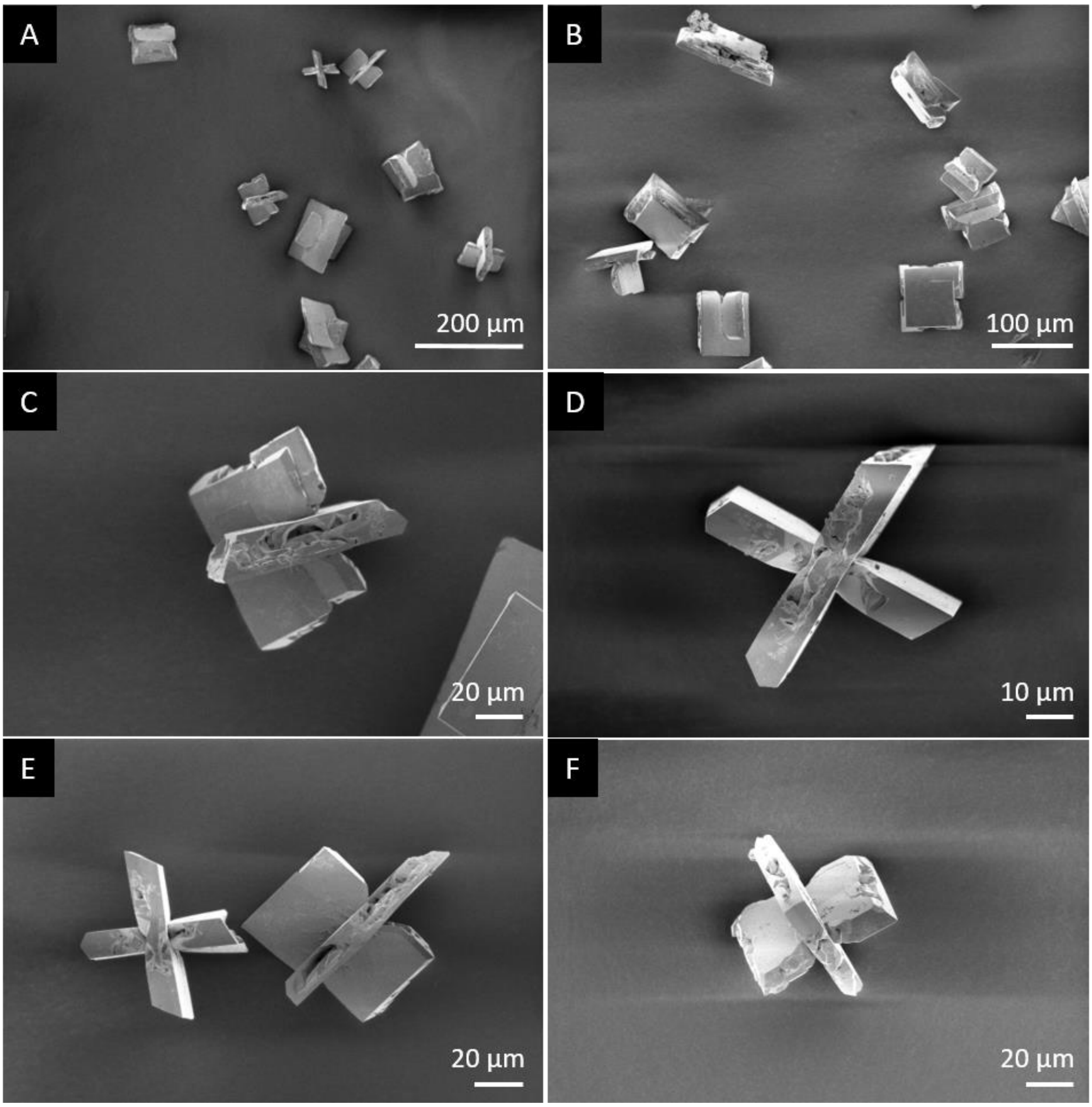

Figure S3. Synthesis of SnO microstructures in the absence of peptide additives. Overview micrographs (A, B) and single SnO microstructures form different experiments (C - F) under the same reaction conditions.

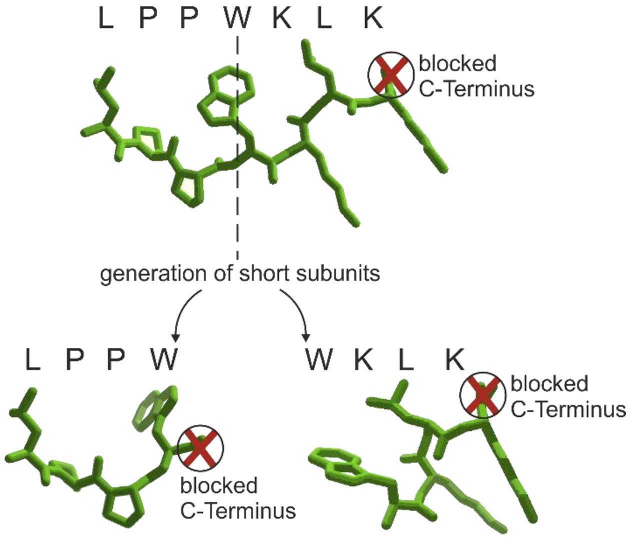

Figure S4. Schematic description of the construction of three solid state synthesized peptides based on the sequence of SnBP01 for the mineralization study of tin(II) oxide microstructures. Peptide SnBP01 (LPPWKLK), peptide SnBP01-1 (LPPW) and peptide SnBP01-2(WKLK). In phage display the peptides are connected by the C-Terminus to the minor coat protein p3, therefore the C-terminus of the synthezised peptides was inactivated by amidation. Structure created with Arguslab (http://www.arguslab.com) and refined with Foldit (https://fold.it/portal/).

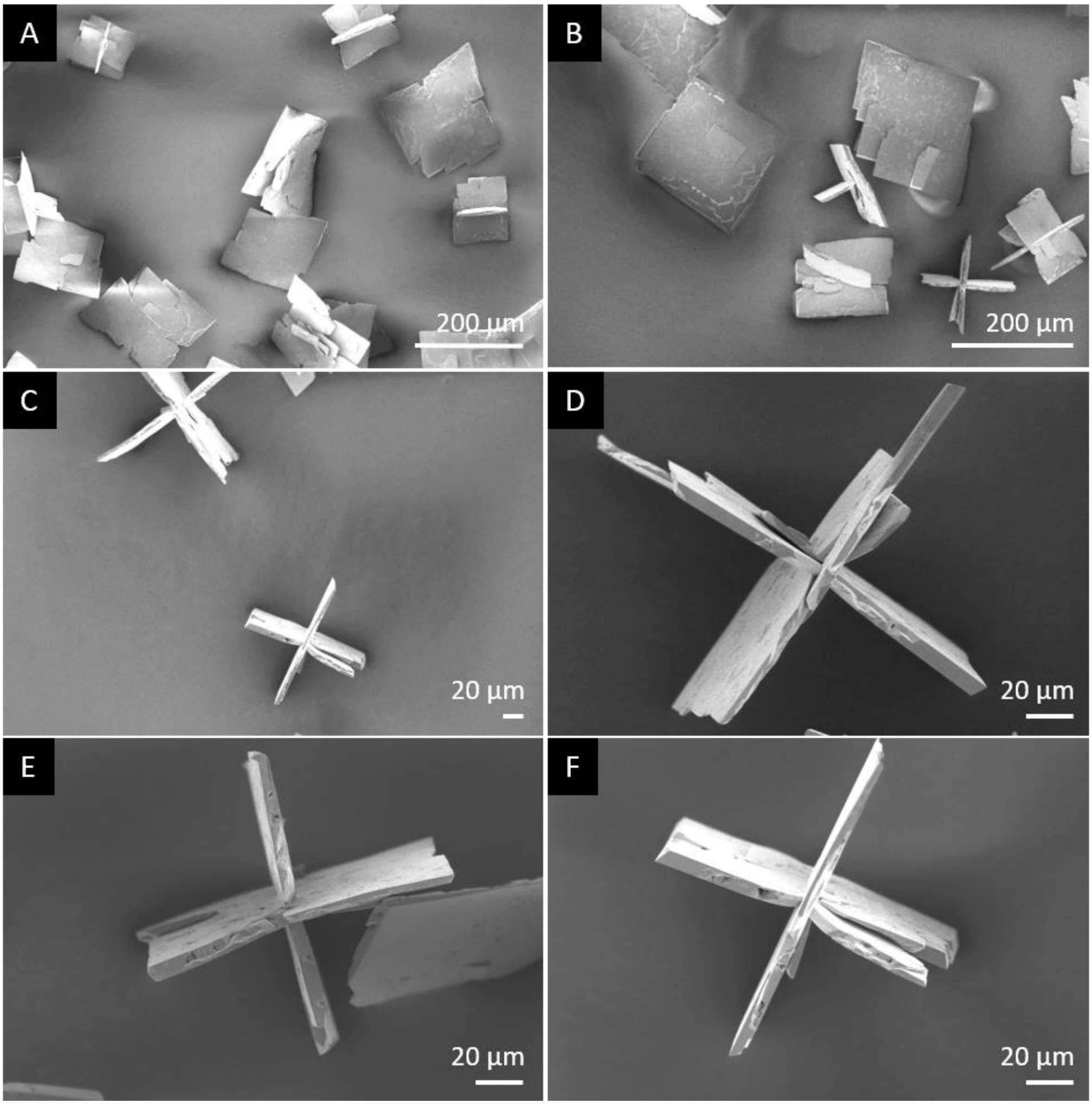

Figure S5. Synthesis of SnO microstructures in the presence of peptide segment SnBP01-2 (WKLK). SnO microstructures were mineralized in the presence of 1 nM peptide. Overview micrographs (A, B) and single SnO microstructures form different experiments (C - F) under the same reaction conditions.

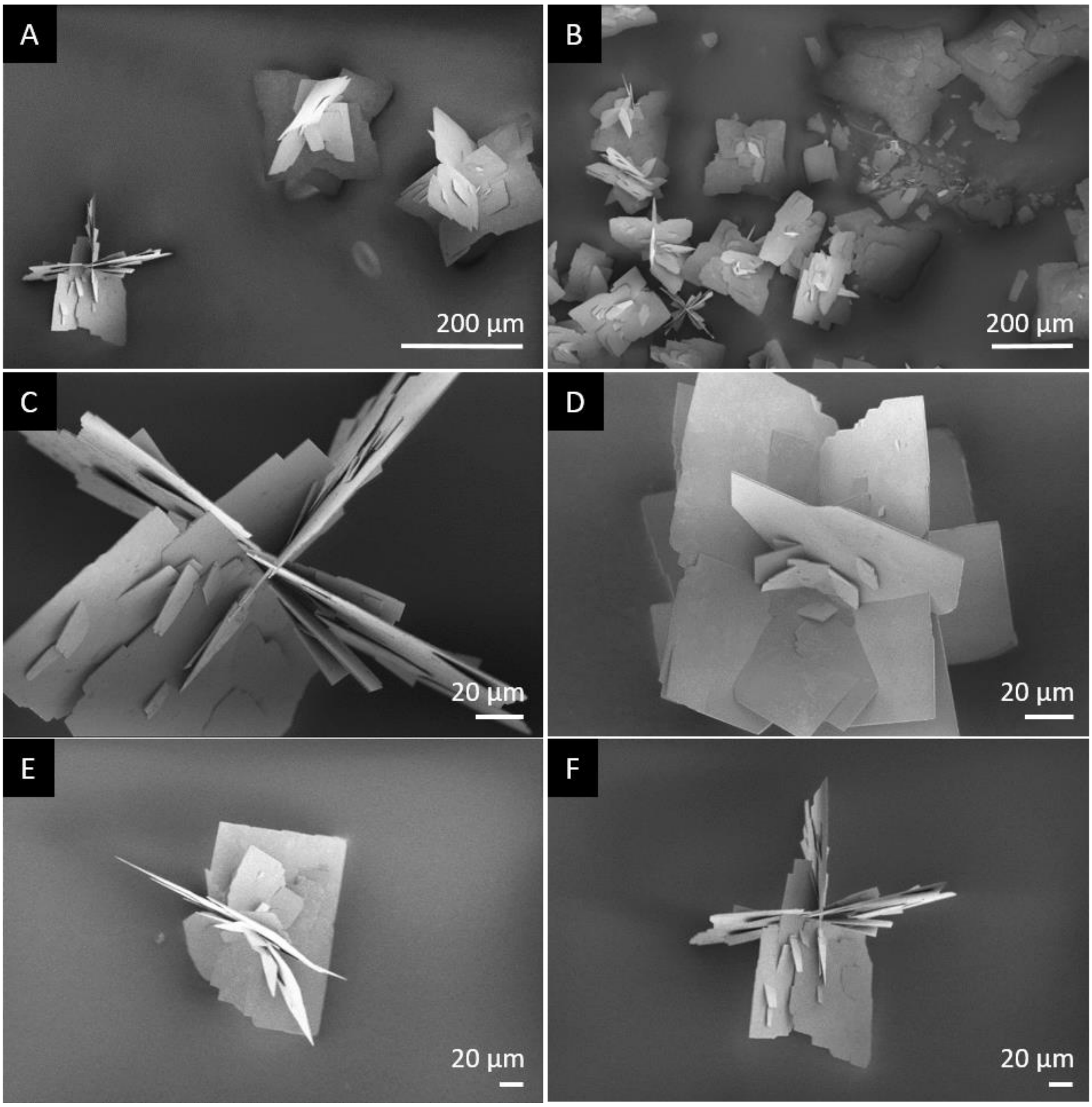

Figure S6. Synthesis of SnO microstructures in the presence of peptide segment SnBP01-1 (LPPW). SnO microstructures were mineralized in the presence of 1 nM peptide. Overview micrographs (A, B) and single SnO microstructures form different experiments (C - F) under the same reaction conditions.

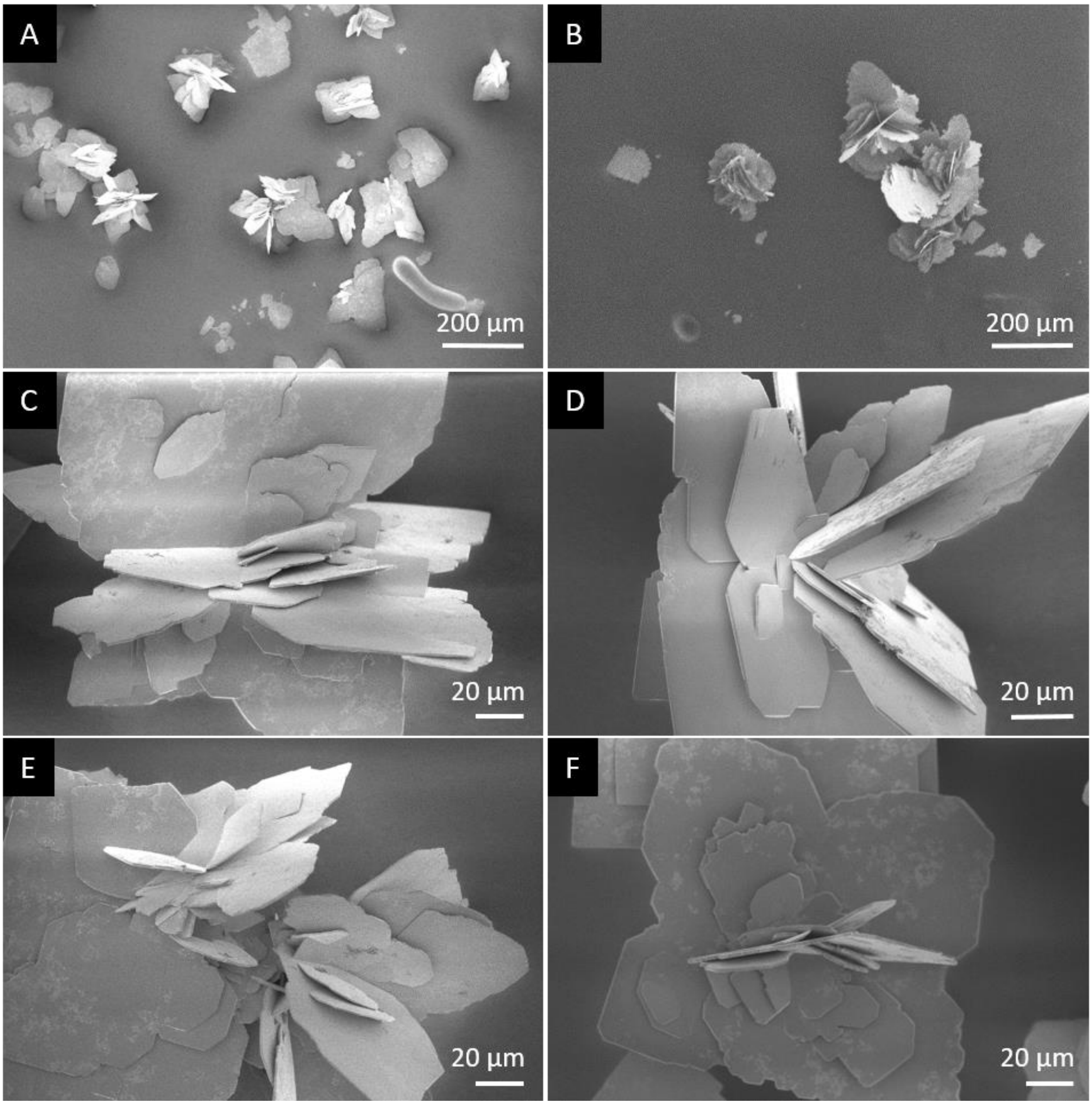

Figure S7. Synthesis of SnO microstructures in the presence of peptide segment SnBP01 (LPPWKLK). SnO microstructures were mineralized in the presence of 1 nM peptide. Overview micrographs (A, B) and single SnO microstructures form different experiments (C - F) under the same reaction conditions.
